# Creatine supplementation and resistance training to preserve muscle mass and attenuate cancer progression (CREATINE-52): a protocol for a double-blind randomized controlled trial

**DOI:** 10.1186/s12885-024-12260-3

**Published:** 2024-04-18

**Authors:** Adriana M Coletta, Lea Haverbeck Simon, Kelsey Maslana, Sarah Taylor, Kish Larson, Pamela A Hansen, Vinay Mathew Thomas, Cornelia M Ulrich, Manish Kohli, Jonathan Chipman, Umang Swami, Sumati Gupta, Benjamin L Maughan, Neeraj Agarwal

**Affiliations:** 1 Department of Health and Kinesiology, University of Utah, Salt Lake City, UT USA; 2 Cancer Control and Population Sciences Program, Huntsman Cancer Institute, University of Utah, Salt Lake City, UT USA; 3 The Huntsman Cancer Institute, University of Utah, Salt Lake City, UT USA; 4 Division of Physical Medicine and Rehabilitation, Salt Lake City, UT USA; 5 Department of Internal Medicine, University of Utah, Salt Lake City, UT USA; 6 Division of Medical Oncology, Huntsman Cancer Institute, University of Utah, Salt Lake City, UT USA; 7 Department of Population Health Sciences, University of Utah, Salt Lake City, UT USA; 8 George E Whalen Department of Veterans Affairs Medical Center, Salt Lake City, UT USA

**Keywords:** Telehealth exercise, Metastatic prostate cancer, Creatine monohydrate, Clinical trial

## Abstract

**Background:**

Muscle mass is important for metastatic prostate cancer survival and quality of life (QoL). The backbone of treatment for men with metastatic castration sensitive prostate cancer (mCSPC) is androgen deprivation therapy (ADT) with an androgen signaling inhibitor. ADT is an effective cancer treatment, but it facilitates significant declines in muscle mass and adverse health outcomes important to mCSPC survivors, such as fatigue, and reductions in physical function, independence, insulin sensitivity, and QoL. In *non-metastatic* CSPC survivors, resistance training (RT) preserves muscle mass and improves these related health outcomes, but the biggest barrier to RT in CSPC survivors of all stages is fatigue. Creatine monohydrate supplementation coupled with RT (Cr + RT) may address this barrier since creatine plays a critical role in energy metabolism. Cr + RT in cancer-free older adults and other clinical populations improves muscle mass and related health outcomes. Evidence also suggests that creatine supplementation can complement cancer treatment. Thus, Cr + RT is a strategy that addresses gaps in survivorship needs of people with mCSPC. The purpose of this parallel, double-blind randomized controlled trial is to test the effects of 52-weeks of Cr + RT compared with placebo (PLA) and RT (PLA + RT) on muscle mass, other related health outcomes, and markers of cancer progression.

**Methods:**

We will carry out this trial with our team’s established, effective, home-based, telehealth RT program in 200 mCSPC survivors receiving ADT, and evaluate outcomes at baseline, 24-, and 52-weeks. RT will occur twice weekly with elastic resistance bands, and an established creatine supplementation protocol will be used for supplementation delivery. Our approach addresses a major facilitator to RT in mCSPC survivors, a home-based RT program, while utilizing a supervised model for safety.

**Discussion:**

Findings will improve delivery of comprehensive survivorship care by providing a multicomponent, patient-centered lifestyle strategy to preserve muscle mass, improve health outcomes, and complement cancer treatment (NCT06112990).

## Background

Declines in skeletal muscle mass are associated with poor cancer survival outcomes, especially among individuals living with metastatic castration-sensitive prostate cancer (mCSPC) [1–7]. For example, among persons with mCSPC a 1-unit decrease in psoas muscle ratio (1 cm^3^/cm), as measured by computerized tomography scan, is linked with a 45% increased risk of mortality [3]. Androgen deprivation therapy (ADT), with or without androgen signaling inhibitors, is effective at improving prostate cancer-specific mortality and is the principal treatment for this disease; however, ADT facilitates significant declines in skeletal muscle mass after just 12-weeks of therapy [8, 9]. Reductions in skeletal muscle mass enable considerable unfavorable changes in health outcomes important to mCSPC survivors and linked with overall survival such as increases in fatigue and fat mass, and declines in physical function, independence, insulin sensitivity, and quality of life [10–12]. Strategies to preserve muscle mass and improve health outcomes and progression free survival are needed for mCSPC survivorship [7, 13].

Engagement in resistance training (RT) exercise twice weekly, activating all major muscle groups, is recommended for individuals living with all stages of prostate cancer to attenuate cancer treatment-related side-effects and improve survival [14]. There is a large body of evidence among *non-metastatic* CSPC survivors receiving ADT that suggests that 12-weeks of in-person, supervised RT, twice weekly, utilizing free weights or resistance machines is safe and feasible to preserve muscle mass, improve fatigue, physical function, strength, and insulin sensitivity, and reduce fat mass [15–21]. Yet only three of these trials included a small portion of individuals living with metastatic disease (*n* < 30) [15, 19–21]. Furthermore, only one RT trial was 52-weeks in length and included a small portion of the sample as mCSPC survivors (*n* = 8) [20, 21]. Collectively, this information presents a gap in our understanding of how longer engagement in RT (e.g., 52-weeks) impacts outcomes in mCSPC survivors receiving ADT.

Adherence to RT recommendations is low among mCSPC survivors receiving ADT [22, 23]. We suspect that RT engagement can be improved with the implementation of a patient-centered approach. The prominent facilitator and barrier to RT among cancer survivors, including mCSPC survivors on ADT, is home-based exercise and fatigue, respectively [24–27]. Work is needed utilizing a more accessible and inexpensive approach to RT delivery, such as use of elastic resistance bands and a home-based program, and a strategy to combat fatigue to enable exercise. Regarding use of elastic resistance bands, a recent systematic review and meta-analysis observed comparable results in muscular strength gains among apparently healthy individuals when comparing supervised, in-person RT programs with elastic resistance bands to programs utilizing weights (e.g., free weights or weight machines) [28]. Work from our group has demonstrated utility in an RT program primarily utilizing elastic resistance bands to attenuate cancer treatment-related side effects among cancer survivors of varying types and stages, including prostate cancer [29]. Furthermore, work from our group has demonstrated feasibility and preliminary efficacy in improving fatigue, among other treatment-related side effects such as muscle mass, physical function, etc., when delivering a 12-week, home-based, supervised via telehealth RT program, with or without creatine monohydrate supplementation, that utilized elastic resistance bands among mCSPC survivors receiving ADT (NCT03987217).

**Fig. 1:**
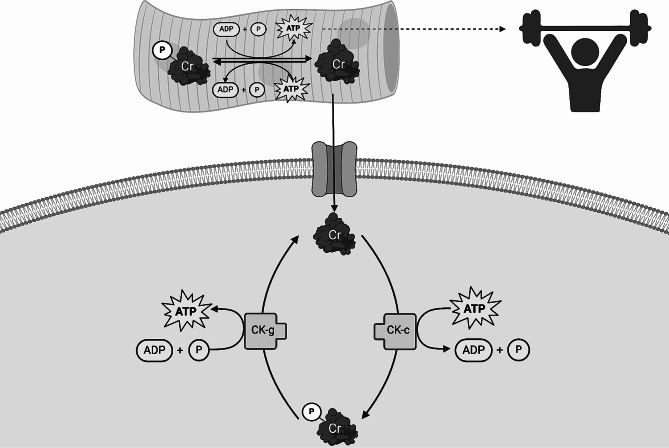
Creatine’s Role in Energy Metabolism. Creatine (Cr) combines with free inorganic phosphate to form phosphocreatine (Cr-P). When adenosine triphosphate (ATP) undergoes degradation into adenosine diphosphate (ADP) and inorganic phosphate (P) to supply free energy for activities, such as exercise, phosphocreatine serves as a buffer for the regeneration of ATP. The generated ATP then undergoes degradation to provide energy and the cycle continues. Additionally, creatine enters the cytosol through a creatine transporter. In the cytosol, creatine, along with its related cytosolic and glycolytic creatine kinase (CK-c and CK-g, respectively) isoforms, plays a pivotal role in preserving glycolytic ATP levels, regulating the cytosolic ATP/ADP ratio, and facilitating cytosolic ATP consumption for glycolysis

The addition of creatine monohydrate supplementation to an RT program can attenuate fatigue, the prominent barrier to RT among mCSPC survivors. Creatine is part of the phosphagen system and plays a critical role in energy metabolism [30]. Creatine monohydrate supplementation increases the availability of creatine and phosphocreatine in the skeletal muscle, which facilitates greater buffering of ATP and enhances exercise capacity and training volume, amplifying RT adaptations (Fig. 1) [30, 31]. Creatine monohydrate supplementation also reduces levels of inflammatory markers associated with severe muscle loss [6]. In addition, creatine monohydrate supplementation may have complementary effects to cancer treatment. Among preclinical models of varying cancer types, including prostate cancer, creatine and its analogs (e.g., cyclo-creatine) have exhibited the inhibition of tumor growth and proliferation [32–37]. In an 8-week trial comparing creatine supplementation to placebo among individuals living with stages III and IV colorectal cancer receiving chemotherapy, a significant increase in phase angle, a marker of body composition measured by bioelectrical impedance, was observed in the creatine group but not placebo [38]. Increases in phase angle indicate improved cellular integrity, permeability, and muscle mass, and have been linked with cancer survival [39, 40].

The purpose of this double-blind, placebo-controlled, randomized controlled trial is to test the efficacy of 52-weeks of a personalized, home-based, supervised via telehealth, RT program with creatine monohydrate supplementation (Cr) or placebo (PLA; Cr + RT -vs- PLA + RT), on changes in muscle mass in individuals living with mCSPC receiving ADT (*n* = 200). We will also test the intervention’s effect on other health outcomes important to mCSPC survivors and mCSPC survivorship: fatigue, physical function, strength, independence, insulin sensitivity, fat mass, and quality of life. Further, we will test the effects of creatine monohydrate supplementation use on changes in markers of cancer progression.

## Methods

### Research design & participant population

#### Research design

This parallel, double-blind randomized controlled trial, that is 52-weeks in lengths, will randomly assign 200 mCSPC survivors receiving ADT to Cr + RT or PLA + RT with randomization in blocks of size four (two randomized to Cr + RT and two randomized to PLA + RT within each consecutive group of four subjects), stratified by age (< 70 years vs. ≥ 70 years) and ECOG performance status (0,1,2). To preserve the blind, only the Investigational Drug Services Pharmacy (IDS) personnel, the Research Compliance Office, and the Data Safety Monitoring Committee at Huntsman Cancer Institute (HCI) will know treatment assignments. Access to unblinded data/documents will be controlled by the pharmacy to ensure the blind is maintained. In the case of an emergency, AMC and NA (mPIs) and the participant’s treating medical oncologist have the sole responsibility for determining if unblinding of a participant’s treatment assignment is warranted. If unblinding is warranted, the investigator will contact IDS for treatment assignment.

**Fig. 2:**
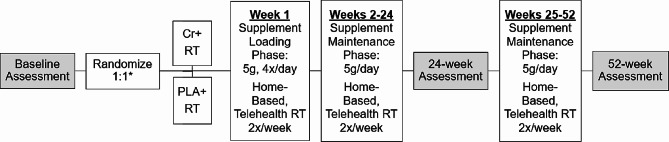
Study Schematic. *Randomization will be in blocks of size four (two per group), stratified by age (< 70 years vs. ≥ 70 years) and ECOG performance status (0,1,2). Cr + RT: Creatine monohydrate supplementation & Resistance Training; PLA + RT: Placebo and Resistance Training; g: Grams

At baseline, completion of 24- and 52-weeks (end-of-study), we will assess muscle mass, fatigue, physical function, independence, insulin sensitivity, fat mass, and quality of life, and collect plasma for cell-free DNA analysis. We will also extract standard of care Prostate-Specific Antigen measurements from the medical record at these time points. All participants will be instructed to continue their typical diet and medication regimens. Figure 2 provides a schematic of the study design, intervention, and assessment time points. This study was approved by the University of Utah Institutional Review Board (IRB_00168125). The trial was registered in ClinicalTrials.gov (NCT06112990) on November 2, 2023 and is currently open to enrollment. Protocol amendments will be made as needed following standard procedures.

#### Participant population

Participants will be recruited from the genitourinary medical oncology clinics at HCI at the University of Utah in Salt Lake City, Utah. The study team’s clinical research coordinators, ST and LHS, will be responsible for obtaining informed consent, carrying out screening procedures, and enrolling participants. Table 1 lists key eligibility criteria. Please see ClinicalTrials.gov registration for a complete list of eligibility criteria (NCT06112990).

**Table 1:** Key Eligibility Criteria

Inclusion Criteria
1. Subject age ≥ 18 years old
2. Metastatic castration-sensitive prostate cancer patients who have not met standard criteria for disease progression on current systemic therapy
3. Currently treated with surgical castration or medical castration with GnRH agonists/antagonists, and/or an androgen receptor pathway inhibitor (e.g., abiraterone, enzalutamide, apalutamide, darolutamide) ^*^
4. ECOG Performance Status ≤ 2
5. Not currently adhering to national physical activity guidelines for resistance training, as defined as participating in structured resistance training ≥ two days per week.
6. Regular access to an electronic device with internet service and ability for video calls
7. Access to an active MyChart account or the willingness to create an account for the purposes of the trial
8. Willingness to engage in a home-based resistance exercise program two days per week
9. Willingness to take creatine monohydrate supplementation or placebo for the duration of the 52-week trial and to avoid taking additional creatine-containing supplementation or other nutritional supplementation during the study period
10. Willingness to complete and submit weekly supplementation logs to study personnel throughout the duration of the 52-week study via email, text, in person, or verbally verified over the phone
11. Willingness to complete three in-person assessment sessions (baseline, 24-, and 52-weeks)
12. Able to provide informed consent and willing to sign an approved consent form that conforms to federal and institutional guidelines
Exclusion Criteria
1. Treatment with cytotoxic chemotherapy within 12 weeks prior to enrollment
2. eGFR < 30 ml/min/1.73m^2^

GnRH- Gonadotropin hormone-releasing hormone

* Must have started the current regimen at least 12 weeks prior to enrollment

### Intervention

#### Resistance training program

We will utilize HCI’s established, effective, hospital-based exercise oncology program, the Personal Optimism With Exercise Recovery (POWER) program, which is run on-site at HCI’s Wellness and Integrative Health Center. Details of the program are described elsewhere [29]. Briefly, POWER consists of a comprehensive physical and medical assessment carried out by a team of physical medicine and rehabilitation physicians and certified exercise physiologists, with expertise in cancer exercise training. The comprehensive assessment consists of testing for muscular strength and endurance, physical function, mobility, balance, and reviewing the participant’s cancer history, presence of other chronic diseases, and documented treatment-related side effects. Information gathered from these assessments is used to develop a personalized RT program including 12 exercises, focusing on all major muscle groups. For this trial, the personalized RT program will be based on data collected at the baseline assessment. To ensure each participant is able to progress as able throughout the duration of the 52-week RT program, they will be provided with “beginner” and “advanced” sets of elastic resistance bands at the baseline assessment. Exercises will progress with a periodization model as recommended by the American College of Sports Medicine [14, 41]. At the 24-week assessment, for each participant, revisions to the RT program will be made based on data collected at that assessment and progression in training. At the 52-week assessment (end of study), a written RT program will be provided to the participant for continued exercise on their own post-study.

At the baseline assessment, information about space availability to exercise in each participant’s home will be gathered to ensure feasibility of the prescribed exercise program within the supervised, telehealth mode of RT program delivery. Additionally, an orientation to the telemedicine platform will be provided as needed during the baseline assessment. The University of Utah’s established telemedicine platform, EPIC Systems Corporation© (Verona, WI) MyChart® application, a HIPAA-compliant software that is run through EPIC, will be utilized for the supervised, telehealth program delivery. Participants can access and use MyChart from any device that has a camera and the ability to connect to the internet. For study participants who do not have access to a device with a camera and internet connection, an iPad will be provided for use throughout the duration of the 52-week trial. During each exercise session, the exercise physiologist supervises the participant to ensure he is completing his RT program correctly, coach the participant through the workout, and record the participant’s compliance to each exercise (e.g. number of sets and repetitions completed for each exercise) within the medical record.

#### Creatine monohydrate & placebo supplementation

Creatine monohydrate supplementation will be provided by NutraBio Labs Inc (Middlesex, NJ) and shipped directly to the HCI IDS Pharmacy from NutraBio Labs for study disbursement. NutraBio Labs manufactures all products in compliance with FDA regulations outlined in the Title 21 Code of Federal Regulations Part 111, Current Good Manufacturing Practice for Dietary Supplements [42]. To ensure product purity and transparency in nutrition labeling, in-house and third-party testing of products is carried out and results from these tests reported publicly (checkmysupps.com). The placebo supplementation will consist of a flavorless dextrose powder developed by HCI IDS Pharmacy. Both creatine monohydrate and placebo supplementation will be stored at HCI IDS pharmacy. Quality control of the supplements (e.g., temperature and humidity levels of environment the supplement is stored in) will be monitored regularly by IDS pharmacy following standard procedures outlined in federal regulation for pharmacy quality control checks. These procedures are carried out to ensure purity and patient safety of supplement use. Additionally, all supplements will be repackaged by the HCI IDS pharmacy team to mask the identity of supplements from participants to protect the blinding of the study protocol. Standard procedures per pharmacy federal regulations will be followed for repackaging.

Participants will receive supplementation (creatine or placebo) at baseline and 24-week assessments and retrieve their supplementation at the HCI IDS Pharmacy. Upon retrieval, a pharmacist will verbally review directions for supplement administration with the participant along with providing an information sheet that recapitulates the directions verbally provided. Regardless of group the participant is randomized to, directions for supplementation use are the same, and based on a well-established protocol [30]. Week 1 is considered the loading phase. Here participants will be instructed to consume five grams of supplementation four times daily, with each dose consumed with 16 fluid ounces of a carbohydrate-containing beverage. The loading phase is established with demonstrated safety in apparently healthy and clinical populations [30, 38, 43, 44], including data from our group which demonstrates safety among men living with mCSPC on ADT (NCT03987217). Weeks 2–52 is considered the maintenance phase. Here participants will be instructed to consume five grams once per day with 16 fluid ounces of a carbohydrate-containing beverage. Creatine monohydrate supplementation contains zero calories per five gram serving. The placebo, which is dextrose, contains 20 calories per five gram serving. For participants who are insulin resistant, a member of the study team will help strategize ways to fit the supplement in his diet. This dietary guidance will be supervised by AMC, who is a Registered Dietitian with clinical experience in diabetes care.

#### Monitoring compliance to intervention procedures

Resistance training program compliance will be monitored and recorded in real time throughout each RT session. Additionally, study personnel will monitor compliance to the number of RT sessions completed per week. Participants will be provided and instructed to complete a daily supplementation log, and email this log to the study team weekly. If an email is not received, a member of the study team will call participants as a reminder to send the log or, if more convenient for the participant, verbally verify and record adherence to supplementation for that week. If a participant misses one week of RT for no given reason, or is non-adherent to the supplementation protocol, study personnel will call these individuals. Unused supplement containers will be returned to the HCI IDS Pharmacy at the 24-week and 52-week assessments for reconciliation.

#### Guidelines for exercise and supplementation dose interruptions and modifications

If a dose of supplementation is missed, it will not be made up for. Additionally, missed RT sessions, planned or unplanned, will not be made up for. The study will utilize the National Cancer Institute’s Common Terminology Criteria for Adverse Events version 5.0 to report adverse events and serious adverse events. If a participants reports excessive soreness of injury from his individualized RT program, the dose of RT will be modified accordingly and modifications associated with adverse events will be recorded in the adverse event log. Regarding supplementation, dose modifications during the loading phase (week 1) secondary to known adverse events from taking creatine supplementation (e.g., gastrointestinal distress) or toxicity felt by the treating oncologist to be significantly related (possibly, probably, or definitely) to supplementation will be reduced to half the recommended amount. Dose modifications during the maintenance phase (weeks 2–52) consist of half the recommended amount until symptoms resolve. Then participants should resume the normal dosing.

#### Data and safety monitoring, and data availability

Study data, except for questionnaire data, will be managed via Advarra® Clinical Trial Management System, which is a HIPAA-compliant platform for building and managing clinical trial databases. Questionnaire data will be managed via REDCap for ease of dissemination and study logistics. This trial will be overseen by HCI’s Data and Safety Monitoring committee (DSMC), which is a committee that functions independently from the institution’s Protocol Review and Monitoring Committee and the Institutional Review Board. The goal of the DSMC is to ensure investigator-initiated trials at HCI are conducted safely, in adherence with regulations on the local and federal level, and to ensure the accuracy, validity, and integrity of study data. The DSMC consists of physician scientists, individuals from other disciplines with experience and knowledge in clinical trial methodology, and cancer biostatisticians. The DSMC assigns medical monitors per trial. This trial is classified as low risk and will be audited annually. Additionally, HCI’s research compliance office will conduct annual, routine audits on this trial, along with an initial audit after the enrollment of the first participant on trial.

Upon completion of data collection, a de-identified dataset will be made available as a CSV data file within the University of Utah’s Research Data Repository, The Hive. (https://hive.utah.edu/). The public can access data stored in The Hive, and the policies and procedures in place for data access to qualified researchers are consistent with the most recent NIH data sharing policies and applicable laws and regulations. Trial results will be disseminated through presentation at international conferences and publication in peer-reviewed journals.

### Assessment procedures & study outcomes

Participants will be instructed to refrain from strenuous exercise at least 24 h before each scheduled assessment session, fast for at least eight hours prior to the blood draw associated with each session, and follow their usual diet and medication regimen. Table 2 outlines study outcomes and procedures.

**Table 2:** Study outcomes and assessment procedures

Outcome	Procedure
Muscle Mass*	Whole-body DXA Scan
Fatigue	FACIT-Fatigue Questionnaire
Physical Function	PROMIS Physical Function Questionnaire
	Short Physical Performance Battery
Strength	Handgrip Strength Test
Independence	Katz Index of Independence in Activities of Daily Living
Insulin Sensitivity (HOMA-IR)	Calculated from measured fasting insulin & glucose**
Fat Mass	Whole-body DXA scan
Serum PSA	Blood Draw
cell-free DNA	

*Primary outcome; **Fasting insulin and glucose assessment requires a blood draw

#### Body composition

A whole-body dual-energy x-ray absorptiometry (DXA) scan will be used to measure lean mass (in grams) and other components of body composition (e.g., fat mass, body fat percentage, appendicular lean mass, fat free mass, visceral fat, etc.). The APEX version 4.0 software within the Hologic Discovery A QDR series DXA system (Waltham, MA) will be used following standard procedures for testing and quality control calibration prior to testing [45, 46].

#### Fatigue

The Functional Assessment of Chronic Illness Therapy-Fatigue (FACIT-Fatigue) version 4 will be used to evaluated fatigue via a score assigned upon completion of the questionnaire. FACIT-Fatigue questionnaire consists of 13 items and includes a reliability coefficient of about 0.90 [47].

#### Physical function

Both objective and subjective measures will be used to evaluate changes in physical function throughout the duration of the 52-week trial. The short physical performance battery will be used as an objective measure to evaluate physical function [48, 49], and will be carried out within the physical assessment portion of the POWER program assessment. The short physical performance battery used for this trial consists of the following three tests: 4-meter walk test, standing balance, and 5-time chair stand test. Participants will receive a score for each test within the battery and a total score for all three tests. Additionally, the PROMIS physical function short form 6b will be used as a subjective measure of physical function. This questionnaire includes six items, and an average reliability coefficient is 0.95 [50].

#### Whole-body strength

Handgrip strength will be evaluated as a measure of whole-body strength, in kg, with use of a portable, hydraulic dynamometer (Jamar 5030J1). Participants will be instructed to sit upright in a chair, keeping feet flat on the floor, shoulders square, and elbow flexed in a 90-degree angle with their forearm of the testing hand resting on a Table [51]. They will then be instructed to squeeze the instrument as hard as they can for 5 s, repeating two more times with a 2-minute rest between sets. This test will be done on each hand, and the best measurement of the three repetitions on each side will be recorded in the study database.

#### Independence

The Katz Index of Independence in Activities of Daily Living (ADL) questionnaire will be used to assesses independence to complete ADLs via a score. The survey has an interrater reliability coefficient of 0.95 [52]. This is a 6-item questionnaire that covers six domains of independence (bathing, dressing, toileting, transferring, continence, and feeding) and is widely used by clinicians to assess ability to perform ADLs [53].

#### Quality of life

The Functional Assessment of Cancer Therapy- Prostate (FACT-P) version 4 includes prostate cancer-specific questions within the assessment of quality of life, via a score. The questionnaire includes 39 items among the following domains: physical well-being, social/family well-being, emotional well-being, functional well-being, and additional concerns. The reliability coefficient of FACT-P is 0.87–0.89 [54].

#### Insulin sensitivity

Fasting serum glucose and insulin will be measured and the homeostatic model of assessment for insulin resistance (HOMA-IR) will be calculated for each time point [55]. The timing of the participant’s study assessments will align as closely as possible with standard of care (SOC) clinic visits. Blood drawn for research purposes will occur in tandem with clinical orders. In the event the participant’s study period is not closely aligned (e.g., at least 2 weeks) with SOC visits, an additional blood draw will need to occur and will be carried out by the hospital’s diagnostic laboratory. Blood collected for insulin sensitivity evaluation will be transferred immediately to Associated Regional University Pathologists (ARUP) laboratory (Salt Lake City, UT) for processing to serum and analysis. ARUP laboratory is the diagnostic laboratory associated with the University of Utah hospital system and is located adjacent to the University of Utah academic and medical campus. Results for glucose and insulin will be extracted from the medical record by the study team.

#### Markers of cancer progression

Serum Prostate Specific Antigen (PSA) and cell-free DNA (cfDNA) will be measured to evaluate changes in markers of cancer progression. Serum PSA, in ng/mL, is measured as standard of care. Collected blood will be transferred to ARUP for analysis and data will be extracted from the medical record by the study team. Blood for cfDNA analysis will be collected in K2-ethylenediamine tetra-acetic acid anticoagulant collection tubes along with other study blood as described above. After collection, these samples will be transferred to HCI’s Biorepository and Molecular Pathology for processing. These samples will be centrifuged at 2,000 g for 10 min, with a brake speed of 7. Plasma will be aliquoted to cryovials and centrifuged once more at 1,500 g for 5 min. Samples will then be stored at -80^◦^C until batch analysis upon completion of data collection.

Upon completion of data collection, circulating cfDNA will be extracted using Maxwell® RSC 48 as per manufacturer’s specifications and using pre-designated kits per manufacturer, which include the Maxwell® CSC Whole Blood DNA Kit for genomic DNA and the Maxwell® RSC for circulating cfDNA. Extracted circulating cfDNA will then undergo quantity and quality check using Qubit 3.0 Fluorometer (Thermo Fisher Scientific, Waltham, MA, USA) and Bioanalyzer 2100 (Agilent Technologies, Santa Clara, CA, USA). For samples with severe genomic contamination from peripheral blood cells, a size selection will be performed with AMPure XP beads (Beckman Coulter, Brea, CA, USA) to remove large genomic fragments. Samples with a total yield < 5 ng will be considered inadequate for formal analysis or for downstream next generation sequencing methods.

### Statistical analysis

With respect to the primary outcome, we anticipate at least a 2 kg difference between intervention arms (Cr + RT vs. PLA + RT) in baseline to 52 week change in muscle mass based on sustaining, or more likely extending, differences seen in a supervised trial conducted by Chrusch and colleagues [56]. In this trial, 30 older men (mean age 70 + years) were randomized to Cr + RT vs. PLA + RT, and body composition and strength endpoints were compared at 12 weeks [56]. In the creatine arm, the men gained an average of 3.3 kg in muscle mass, while in the placebo arm, the men gained 1.3 kg, corresponding to a 2 kg difference in lean mass change over 12 weeks.

Muscle mass values within individuals are expected to be strongly correlated (after adjusting for time by treatment arm). In Chrusch and colleagues [56] study, repeated measurements of muscle mass over a short time interval, and without an intervening treatment, had an intra-subject correlation of 0.99. At each time point, the standard deviation of muscle mass was approximately 5.8. In our 12-week pilot study, we found a standard deviation of muscle mass of 8.24 kg with an intra-subject correlation between baseline and 12-week measurements of 0.975. We might realistically expect intra-subject correlation (ICC) of at least 0.85, given intervening treatment with RT and potentially creatine as well as a 52-week follow-up period. For a sample size of 200 (100 per arm), if the ICC is 0.85, then the power will be ∼ 84% for a mean difference of 2 kg muscle mass change.

The primary analyses will be conducted from a modified intention-to-treat perspective, with each subject analyzed according to their randomized study arm, irrespective of participation or compliance, as long as they complete their baseline assessments. Changes in muscle mass from baseline to 52-weeks will be compared between the Cr + RT and PLA + RT arms in the context of a linear mixed effects model, based on each subject’s measurements at baseline 24-weeks and 52-weeks, with a random effect for subject, and fixed effects for time, study arm, study arm by time interaction, and a priori anticipated important drivers of changes in muscle mass (age, length of time on ADT, ECOG performance status) at randomization. The effect estimate, and associated Wald test, of the study arm at 52 weeks corresponds to the estimate of the intervention effect (Cr + RT vs. PLA + RT), and its associated hypothesis test.

A few additional analyses are pre-planned to add robustness and clarity of interpretation. One suite of additional analyses will omit the 24-week follow-up measurements from the primary analysis model. Additional analyses will also be conducted omitting the anticipated drivers of responsiveness to intervention from the collection of adjustment variables. Lastly, we plan to conduct a suite a subgroup analyses, accompanied by subgroup by intervention arm interaction tests, for subgroups defined by age, length of time on ADT, baseline muscle mass, RT compliance (≥ 80% of scheduled sessions vs. <80%), and supplement compliance within the Cr + RT arm (≥ 80% of prescribed dose consumed vs. < 80%). Within the Cr + RT arm, compliance to creatine supplementation protocol will be summarized as percentage of prescribed doses overall and by phase (loading vs. maintenance phase), and changes in muscle mass from baseline to 52 and 24-weeks summarized separately for compliance groups (≥ 80% of prescribed dose vs. <80%, and ≥ 80% of scheduled sessions vs. <80%).

Analyses will assume that data is missing-at-random (MAR; data values are independent of the missing data mechanism conditional on the observed data), the most general data-centric missing data assumption [57]. Missing outcome data (e.g. muscle mass) will handled by simply omitting those observations from the mixed effects model, which is valid under MAR. Missing data on covariates (e.g., age, length of time on ADT, and ECOG performance status) will be handled via multiple imputation, which also ensures validity under MAR [58].

Notably, follow-up measurements missing due to patient death will not be MAR, and follow-up measurements missing due to severe declines in patient health may not be MAR either. Throughout, estimates for differences in baseline to follow-up changes in endpoints between the study arms will be conditional on the subset of patients who would have been able to continue on study procedures. While we do not anticipate enough overall survival or progression-free survival events for well-powered comparisons, these endpoints will be compared between study arms in supplemental analyses. Additionally, while we expect both study arms to provide benefits to our patients, the study will be under continuous oversight of an independent Data Safety Monitoring Committee at HCI, which will be monitoring for risks to patients.

Analyses for secondary endpoints (e.g., fatigue, physical function, cfDNA, etc.) will mirror the approaches described above for muscle mass. Additionally, we will examine relationships between changes in markers of cfDNA and PSA and changes in body composition, fatigue, strength, physical function, insulin sensitivity, and quality of life, at both 24 and 52-weeks. These relationships will be examined in the context of linear models adjusted for anticipated drivers of changes in endpoints such as baseline endpoint value, age, length of time on ADT, and ECOG, in the overall sample adjusted for study arm as well as separately for each study arm.

## Discussion

The Creatine-52 trial aims to identify the most effective solution to address the needs of mCSPC survivors receiving ADT. Findings from this trial will build upon previous work from the investigative team, where we demonstrated safety, feasibility, and efficacy of 12-weeks of Cr + RT compared with RT alone to be superior in preserving muscle mass, improving fatigue, physical function, and whole-body strength (NCT03987217). This will be the first study, to our knowledge, to assess longer-term (e.g., 52-weeks) efficacy of Cr + RT compared with PLA + RT on changes in muscle mass, health outcomes, and quality of life; which is important because longer-term solutions that address needs of mCSPC survivors are needed. Furthermore, a current gap in our knowledge is the efficacy of long-term engagement in RT and creatine monohydrate supplementation use in this patient population to improve health outcomes.

Additionally, this approach addresses a major facilitator to RT among mCSPC survivors, implementation of home-based programs [24, 25, 59], while utilizing a supervised model for safety. This approach is translational as it can be adopted by cancer centers nationwide who use an established telemedicine system. Findings from this trial will also improve delivery of comprehensive survivorship care by providing a multicomponent, patient-centered lifestyle strategy to preserve muscle mass, improve health outcomes and quality of life, and complement cancer treatment in this patient population. This patient-centered strategy can be translated on a large-scale because creatine monohydrate supplementation and elastic resistance bands are inexpensive and widely available. Further, our findings will lead to simple recommendations that the oncology care team can provide in clinic (e.g., “Take 5 grams of creatine monohydrate daily, and engage in an RT program twice per week”). If the hospital does not have an associated exercise oncology program, the provider can instruct the patient to consult the American College of Sports Medicine’s Moving Through Cancer Exercise and Rehabilitation program directory to find a local program that fits the patient’s needs [60].

Development of this trial was not only informed by the 12-week trial carried out by the investigative team, but also in coordination with the Salt Lake City Community Prostate Cancer Support Group. Individuals from this group helped the investigative team refine the intervention and assessment procedures. This group will continue to partner with the investigative team throughout the duration of the project by attending bi-annual investigative team meetings. Examples of input provided by the community group include inquiry of space needs at assessment sessions for RT program delivery, and submission of weekly supplementation logs for accountability.

In summary, this trial aims to test a patient-centered approach to a problem of high priority within survivorship care of individuals living with mCSPC. More research related to supportive care interventions in the metastatic setting are needed considering the growing prevalence of individuals living with metastatic cancer [61]. This work also aligns with the most recent NCI Cancer Moonshot priority which is to “improve the experience of people and their families living with and surviving cancer” [62].

## Data Availability

No datasets were generated or analysed during the current study.

## Abbreviations

ADL: Activity of Daily Living
ADT: Androgen Deprivation Therapy
ARUP: Associated Regional University Pathologists
cfDNA: cell-free DNA
Cr: Creatine monohydrate supplementation
Cr + RT: Creatine monohydrate + Resistance Training
DSMC: Data Safety Monitoring Committee
PLA + RT: Placebo + Resistance Training
POWER: Personal Optimism With Exercise Recovery
PROMIS: Patient Reported Outcomes Measurement Information System
PSA: Prostate Specific Antigen
QoL: Quality of Life
RT: Resistance Training
SOC: Standard of Care
DXA: Dual-energy X-ray Absorptiometry
ECOG: Eastern Cooperative Oncology Group
FACIT-Fatigue: Functional Assessment of Chronic Illness Therapy- Fatigue questionnaire
FACT-P: Functional Assessment of Cancer Therapy-Prostate questionnaire
FDA: Food and Drug Administration
HCI: Huntsman Cancer Institute
HIPAA: Health Insurance Portability and Accountability Act
HOMA-IR: Homeostatic Model Assessment for Insulin Resistance
ICC: Intra-subject Correlation Coefficient
IDS: Investigational Drug Services
mCSPC: Metastatic Castration Sensitive Prostate Cancer
MAR: Missing At Random
PLA: Placebo
